# When None of Us Perform Better than All of Us Together: The Role of Analogical Decision Rules in Groups

**DOI:** 10.1371/journal.pone.0085232

**Published:** 2014-01-14

**Authors:** Nicoleta Meslec, Petru Lucian Curşeu, Marius T. H. Meeus, Oana C. Iederan Fodor

**Affiliations:** 1 Department of Organisation Studies and Center for Innovation Research, Tilburg University, Tilburg, The Netherlands; 2 Cognitrom, Cluj-Napoca, Romania; 3 Department of Psychology, “Babeş-Bolyai” University, Cluj Napoca, Romania; Cajal Institute, Consejo Superior de Investigaciones Científicas, Spain

## Abstract

During social interactions, groups develop collective competencies that (ideally) should assist groups to outperform average standalone individual members (weak cognitive synergy) or the best performing member in the group (strong cognitive synergy). In two experimental studies we manipulate the type of decision rule used in group decision-making (identify the best vs. collaborative), and the way in which the decision rules are induced (direct vs. analogical) and we test the effect of these two manipulations on the emergence of strong and weak cognitive synergy. Our most important results indicate that an analogically induced decision rule (imitate-the-successful heuristic) in which groups have to identify the best member and build on his/her performance (take-the-best heuristic) is the most conducive for strong cognitive synergy. Our studies bring evidence for the role of analogy-making in groups as well as the role of fast-and-frugal heuristics for group decision-making.

## Introduction

Organizations extensively use groups to perform a variety of cognitive tasks [1] and collective decisions are essential for organizational performance [2]. Reliance on groups in social life is built on a strong assumption, namely that the array of information exchanged, explored and integrated in groups enhances decision quality relative to individual choices [3], [4]. Similarly, other species organize and work in collectives in order to enhance their survival chances. For example, homing and migrating birds collectively decide on communal routes that maximize their chances of survival and successful arrival to their destination and swarms of bees and ants collectively choose new nest sites on which their survival depends [5]–[7]. Social interactions unfolding in such collectives shape the emergence of collective choices that transcend a simple aggregation of individual preferences or competencies [8]–[10].

Although groups have the potential to become superior (as interacting collectives) to standalone individuals or simple aggregation of individual actions or competencies, this (emergent) potential is not always realized in real-life situations. Studies stemming from the group synergy literature illustrate not only that groups do not manage to achieve strong cognitive synergy (they fail to perform better than their best individual member [11]–[13]) but sometimes they even have difficulties to achieve weak cognitive synergy (they perform worse than the average individual performance in the group [14], [15]). Obviously, group synergy is a group emergent phenomenon that is rather difficult to achieve in interacting groups [16]. Therefore, understanding the way in which individual choices and competencies are combined and coordinated through social interactions in order to generate superior collective outcomes is of key importance to understanding the emergence of collective cognitive competencies [16], [17].

This paper investigates experimentally how inducement and the nature of decision rules affect group synergy. In line with Kurt Lewin's statement that “you cannot understand a system until you try to change it” [18] and in order to better understand how groups work in their attempt to achieve strong cognitive synergy we test the effect of direct versus analogical ways of inducing two decision rules, namely the collaborative and identify the best decision rule. One way in which groups can increase the quality of their collective choices is to identify their best performing individual and improve its performance. Simulation studies indicate the superiority of the expert rule, that requires the identification of the expert member of the group in comparison to the aggregate rule that requires the ability to pool information from multiple individuals in the group, and call for empirical studies testing this particular prediction in real life groups [19]. However, the literature to date only tested the effects of decision rules that were directly induced and little interest is shown to explore the role of decision rules that are autonomously developed by groups. As groups in modern organizations are increasingly autonomous [20]–[22] and often copy other successful groups [23] or individuals in their environment [24], it becomes highly relevant to contrast the directly induced decision rules with the rules developed by groups through analogy.

### Group cognitive synergy and decision rules

Collective cognitive competencies (e.g., collective intelligence, group rationality) refer to the ability of groups to accomplish collectively things that cannot be achieved by the aggregation of individual (cognitive) efforts. Collective cognitive competencies reflect groups' synergetic cognitive processes [25]. Group synergy is achieved when the collective performance of interacting individuals becomes higher than the performance achieved by a simple combination of standalone group member efforts [25]. Two types of group synergy are discerned in our study: 1) weak cognitive synergy when collective cognitive performance is better than the average performance of group members, and 2) strong cognitive synergy, when collective performance exceeds the performance of the best performing individual in the group [25].

Although previous research shows how social interaction can foster group synergy as an emergent phenomenon [16], groups often have difficulties in becoming better than their best individual member [11], [13] or the average performance of the group members [14], [15], [26]. A number of decision rules have been developed in order to guide group interactions and minimize process losses (e.g., unequal participation, loafing) associated with low performance [27]. Decision rules are prescribed norms that guide the interaction of the group members and influence the way in which information is communicated and integrated in the group. The purpose of this first study is to contrast two such decision rules on the one hand and their way of inducement on the other hand in order to explore which rule (collaborative or identify-the-best) induced in which way (direct or analogical) is the most beneficial for group cognitive synergy.

#### Collaborative vs. identify-the-best decision rules

The collaborative decision making rule has received considerable attention in both human and animal group research [16], [27], [28]. The collaborative decision rule encourages opinion sharing and equal participation of all group members during deliberations. It turns out that external facilitators that encourage the participation of all group members in the task contribute to group decisions that exceed the decision of the best performing member in the group [27]. Given that group members are provided with the opportunity to discuss and contribute with their unique knowledge and expertise, collaborative decision rules are conducive to knowledge integration and foster decision quality. Studies have indicated that although on average, groups did not manage to achieve strong synergy, groups that follow a collaborative decision rule managed to get closer to the rationality of their most rational group member than groups following a consultative rule [16]. Therefore, although the collaborative rule increases the information processing efforts in groups, it also has shortcomings: (1) in absolute terms it has not yet been proved to lead to strong cognitive synergy, and (2) it comes with costs in terms of time and cognitive resources that need to be invested in the group decision.

This study pursues to address these two shortcomings by using heuristics inspired from the ecological rationality view. Heuristics are decision-making strategies that simplify the decision situation and assist decision-makers in making frugal and accurate decisions using rather limited information [24], [29], [30]. The highlight of ecological rationality literature is the less-is-more effect, which illustrates an inverse U-shaped relation between the level of decision accuracy and the amount of information considered. In line with ecological rationality, we argue that a decision rule such as identify-the-best is particularly relevant to cognitive synergy, given that the core of strong synergy lies in groups outperforming its best individual member.

We test the use of a particular heuristic decision rule (identify-the-best), which decreases the information processing demands on groups, and as a consequence fosters the emergence of strong cognitive synergy. Identify-the-best heuristic requires group members to identify the most capable member in the group and to improve his/her performance. This comes close to the take-the-best decision-making heuristic because group members need to search in the group the person with the highest decision accuracy, stop the search when the person is found and adopt that person's decision as the group's decision which is considered further for improvement. Take-the-best heuristic has been proved to be an effective strategy in predicting accuracy as compared to other more complex decision strategies [29], [31].

Given that the identify-the-best rule relies on 1) a simple adaptive decision-making heuristic that does not require groups to draw on a large pool of information when establishing their group decision rule and deciding as a group, and 2) is directly conducive to cognitive synergy we expect it to yield superior outcomes in generating strong cognitive synergy relative to the collaborative decision rule. A simulation study indicates that the expert choice rule (identification of informed individuals) is actually a better decision rule than the aggregate rule (ability to pool information from multiple individuals) in single-shot decisions [19]. The result remains stable even when the probability of groups to fail identifying the best performing group member is accounted for. Therefore, we expect the identify-the-best decision rule to be superior to the collaborative rule for groups in reaching cognitive synergy.

#### Direct vs. analogical inducement

In this study we also manipulate the way in which decision rules are induced. Recent experimental research only explored the effects of directly induced decision rules [16]. Self-managing groups are information processing systems that need to continuously adapt to their environments and they often copy successful work-related practices and processes used by other successful groups [23], [24]. Therefore, decision rules with the potential to foster strong synergy may stem from analogies made with successful groups in the environment. In real organizational groups, normative frameworks guiding interpersonal interactions (e.g., decision rules) can be directly induced through top-down managerial interventions [32]. Nevertheless, in self-managed groups these normative frameworks are often generated autonomously by group members themselves [20].

In the direct inducement condition, group members are directly instructed which rule to follow while in the analogical condition groups have to find their own decision rule while using relevant examples of successful decisions made by individuals or groups. Thus, via analogy with a successful group positioned in a similar decision situation, groups need to construct a viable decision rule for their own group. The analogical manipulation is in fact a form of the imitate-the-successful heuristic. Imitate the successful is a social heuristic that is successfully used not only in humans [29], [33] but also animals [34]. Archer fish for instance is a species that learns a difficult insect hunting technique mainly from extensive observation of the skilled fish who already acquired the technique instead of extensive training or trial-and-error attempts [34].

Given the combination of manipulations (type of rule x way of inducement) we developed two sets of predictions. On the one hand, we expect groups that follow identify-the-best rule via analogy (IBA) to be superior to groups that follow the collaboration rule via analogy (CA). In the IBA condition groups have to establish their own decision rule while imitating a successful case and the successful case in this particular condition employs a decision rule in which the most capable member needs to be identified and his/her performance improved before becoming a part of the group's solution. IBA is thus a condition that reflects a combination of two heuristics: imitate-the-successful and identify-the-best which is particularly adaptive for groups that pursue cognitive synergy. Given that CA involves imitating a decision rule which draws on a large pool of information and which it is not particularly adaptive for the case of group cognitive synergy, we expect it to yield results which are inferior to the IBA condition. On the other hand, we expect the direct inducement to be superior to the analogical one, given that groups are explicitly instructed to use the decision rule offered to them and do not need to derive it analogically, which involves an extra step in the group's process of establishing a decision-making rule.

In summary, we expect that: 1) the level of the group synergy in the collaborative direct condition (CD) exceeds the group synergy in the collaborative analogical (CA) condition, and 2) that the level of group synergy in the identify-the-best direct (IBD) condition exceeds the level of group synergy in the IBA condition.

### Ethics statement

We report the results of two experimental studies, one conducted in Romania and one in The Netherlands. The studies were designed as integrated part of curricular activities. Participants were invited to participate in a group decision exercise as part of a workshop on individual and group decision making (part of an Organizational Behavior course in a Dutch university and part of a Social Psychology course in a Romanian university). Because the exercise is part of curricular activities, it involves no foreseeable risk for the participants. Given that (1) the experiment was conducted as part of class related activities and no risks greater than the risk usually associated with class attendance in higher education programs were involved and (2) according to the Dutch and Romanian ethical guidelines, studies involving filling out short questionnaires that do not involve highly sensitive or embarrassing issues are exempted from ethical committee approval, no ethical committee consent was required for this study. Participants were nevertheless informed verbally and in writing that their questionnaires filled out during this particular workshop would be (anonymous) used for scientific research. All students could access a message placed in the electronic communication system and before the exercise all participants were verbally informed that the results would be used for scientific research and asked for their verbal consent. Moreover, participants were informed that if they experience distress associated with their participation in the exercise, they should notify the teachers immediately and they had the chance to opt out if they decided to do so. No participant however reported any distress associated with the task. This was further confirmed during the debriefing session, where participants referred to the exercise as a valuable and attractive learning experience.

## Study 1

### Methods

#### Participants and procedure

One-hundred-forty-six students enrolled in an introductory course at a Romanian University (Age_mean_ = 20.59, 130 females) participated in the study. The students were informed that they will participate in a decision-making exercise as part of their collaborative learning experience and were debriefed at the end of the exercise. The Winter Survival exercise [35], [36] is a disjunctive decision-making task that has a correct solution. In the task, the participants had to imagine that they have just survived the crash of a small plane in the north of Canada, in extreme cold conditions. Having 12 items at their disposal they had to decide which ones are the most important for their survival. Therefore, the task is to rank-order the 12 items from lowest to highest importance for their survival. In a first step, group members had to rate the objects individually. After performing the exercise individually, the students have been assigned to 48 groups (average group size = 3.04) and were asked to perform the same task in groups. The task order (administered first to individuals and then to groups) comes in line with the conceptual framework of cognitive synergy which attempts to capture the role of interpersonal interactions on the emergence of group cognition. Previous studies investigating the concept of group cognitive synergy have used a similar task succession [13], [16], [37].

Finally, at the end of the exercise, participants compared their individual performance scores with the group scores and reflected upon the impact of decision rules upon group dynamics. Of particular interest for discussions were groups that managed to become better than the best performing group member and groups that failed. Social and organizational implications of group decision making have also been discussed as part of the debriefing.

#### Manipulations

In the current study we crossed two manipulations (decision rule and type of inducement), each with two possible conditions. We have used a between-group design. The 48 groups have been allocated to one of the four possible conditions: 12 groups to identify-the-best direct (IBD) condition, 11 groups to collaborative direct (CD) condition, 13 groups to identify-the-best analogical (IBA) condition and 12 groups to the collaborative analogical (CA) condition. In the direct inducement conditions, groups have been asked to employ either the method of group collaboration (CD) or the decision rule of identifying the best performing group member (IBD). The method of group collaboration [37] involves that ranking for each of the 12 survival items must be agreed upon by each group member before it becomes a part of the group decision. The decision rule of identifying the best performing group member involves that group members must strive to identify who is the most capable member in the group and then try to improve his/her performance. For the first two conditions (IBD and CD), the two decision rules have been directly communicated to the groups. For the last two conditions (IBA and CA) group members had to follow the same rules of collaboration and identifying-the-best, but this time the inducement has been made via analogical scenarios. In condition IBA, before solving the task groups had to read for 5 minutes a scenario that described the behavior of ant colonies in food searching. The scenario contained an example in which the ants were explicitly identifying the most successful ant in the colony (the ant who managed to find a food source) and working on improving its performance (accentuating the pheromone trail on the path leading to the food source so that even more ants would be able to identify the path to food) (follow- the- best heuristic) [38]. While drawing on the ants' scenario, groups were asked to discuss for 15 minutes and elaborate their own group decision rule which they can further on use in the Winter Survival task (imitate-the-successful heuristic). Thus in the IBA condition we have induced the same decision rule of identifying-the-best in an analogical rather than a direct way. We have applied the same logic to the CA condition, in which we induced the idea of collaboration via a scenario of bees which were deciding for choosing a new nest. The scenario was designed in such a way that it suggested the idea of collaboration as being crucial for the success of the bee colony [28], [39] After reading the bees' scenario, groups were asked to discuss for 15 minutes and elaborate their own successful group decision rule which they can further use for the task.

#### Measurements

Performance scores (as accuracy measures) were obtained by comparing individual and group rankings with the expert rankings. The absolute differences between individual and group rankings on the one hand and expert rankings on the other hand have been summed up to obtain a performance indicator for both individuals and groups. For the sake of clarity we used recoded raw performance scores in the analyses given that high scores were indicative of low performance and low scores of high performance [13]. Weak cognitive synergy has been computed by subtracting the mean of individual scores in the group from the group score [25]. Positive scores of weak cognitive synergy reflect thus that the group managed to perform better than the average of its group members while negative scores indicate that the group actually performed worse than the average. Strong cognitive synergy has been computed by subtracting the score of the best performing member of the group from the group performance score. Positive scores indicate that the groups managed to outperform their best individual member and negative scores indicate that the groups did not manage to become better than their best individual member.

### Results Study 1

In order to test our hypotheses we ran a GLM multivariate analysis with weak and strong cognitive synergy as dependent variables. Given that in larger groups the levels of participation and thus knowledge integration are lower we have added group size as a covariate in the analysis. Next to this, the maximum score in the group and gender variety have also been added as covariates. The use of maximum score is an attempt to control for lower likelihood of achieving strong synergy when the best performer scores are very high [13], [16]. Group gender composition has been found to be an important predictor of emergent collective cognitive competencies [16], [17]. Therefore we have included gender diversity as a covariate. Gender diversity has been computed with the Teachman's index [40], [41]. Means, standard deviations and correlations of the variables included in the study are presented in Table 1. Descriptive statistics of the manipulations are presented in Table 2.

**Table 1:** Correlation table with descriptive statistics Study 1 (N = 48).

	Mean	*SD*	1	2	3	4
1.Weak cognitive synergy	−0.21	7.03				
2.Strong cognitive synergy	−5.50	7.21	0.91***			
3. Gender variety	0.13	0.26	−0.03	−0.15		
4. Individual maxim score	21.54	5.25	0.13	−0.08	0.06	
5. Group size	3.04	0.28	−0.04	−0.19	−0.07	0.12

Note: ***<.01.

**Table 2:** Descriptive statistics manipulations Study 1.

	Mean		SD		N
	WS	SS	WS	SS	WS/SS
Identify-the-best direct	−2.25	−6.16	6.12	5.93	12
Collaboration direct	−0.98	−7.81	8.72	8.68	11
Identify-the-best analogical	1.76	−3.69	6.98	7.37	13
Collaboration analogical	0.38	−4.66	6.44	6.97	12
Analogical rule	1.10	−4.16	6.62	7.05	25
Direct rule	−1.64	−6.95	7.33	7.25	23
Identify-the-best	−0.16	−4.88	6.76	6.70	25
Collaboration	−0.26	−6.17	7.47	7.81	23

Note: WS =  weak cognitive synergy; SS =  strong cognitive synergy; N =  number of groups.

Our results indicate that there are no significant differences between the collaborative and identify-the-best decision rule F (1, 48) = 0.09, p = 0.75 for weak cognitive synergy nor for strong cognitive synergy F (1, 48) = 0.28, p = 0.59. The simple contrast estimate is t = 0.71, p = 0.75 for weak cognitive synergy and is t = 1.18, p = 0.59 for strong cognitive synergy. The interaction effect between the two types of manipulations is also not significant, F (1, 48) = 0.15, p = 0.69 for weak cognitive synergy and F (1, 48) = 0.005, p = 0.94 for strong cognitive synergy. There are also no differences between the two types of rule inducement, with F (1, 48) = 2.40, p = 0.12 for weak cognitive synergy and F (1, 48) = 3.22, p = 0.08 for strong cognitive synergy. The simple contrast estimates between the analogical and direct condition are t = 3.35, p = 0.12 for weak cognitive synergy and t = 3.85, p = 0.08 for strong cognitive synergy.

### Discussions Study 1 and Introduction Study 2

**Figure 1:**
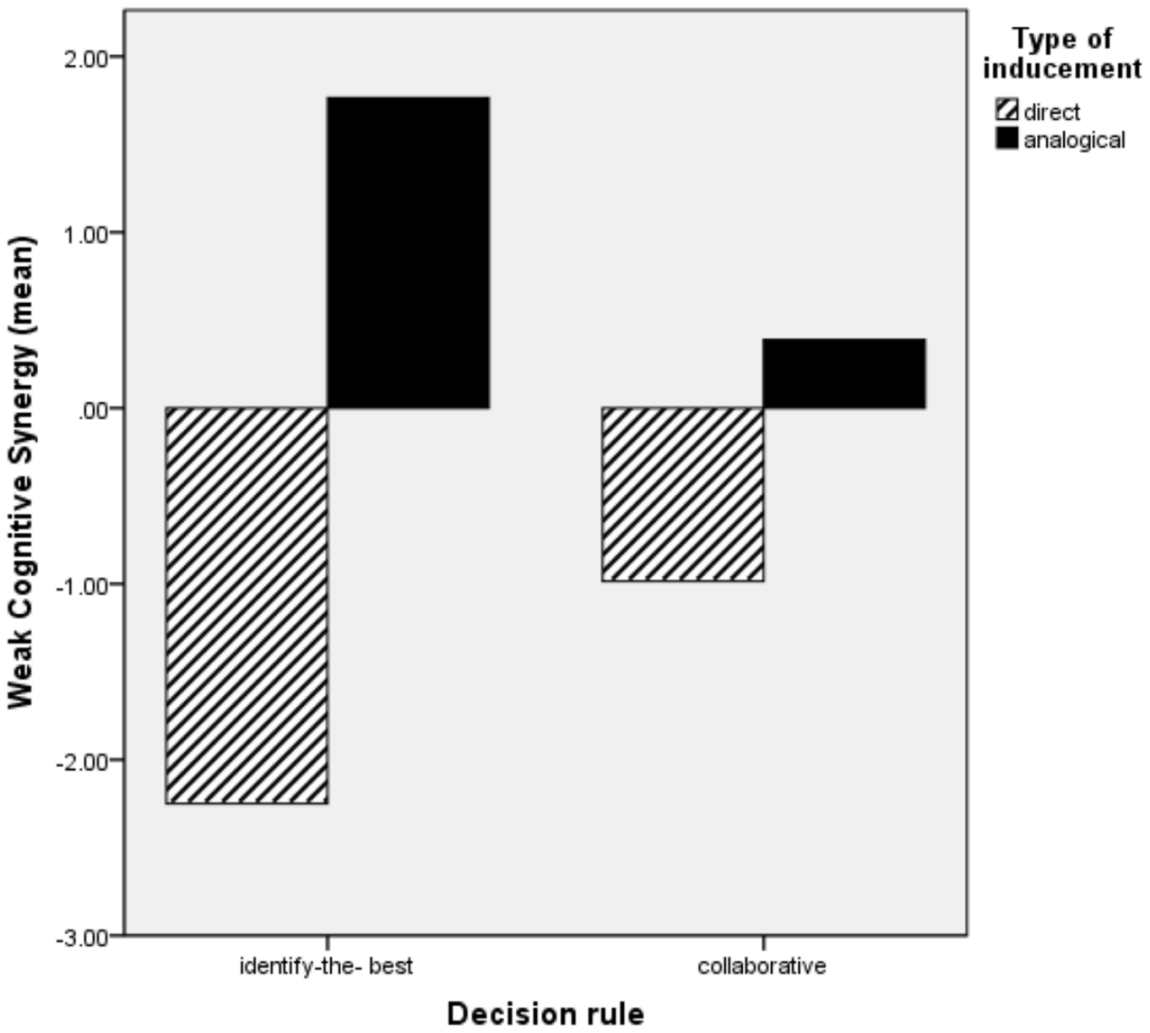
The interaction of decision rule and manipulation inducement on weak cognitive synergy Study 1.

**Figure 2:**
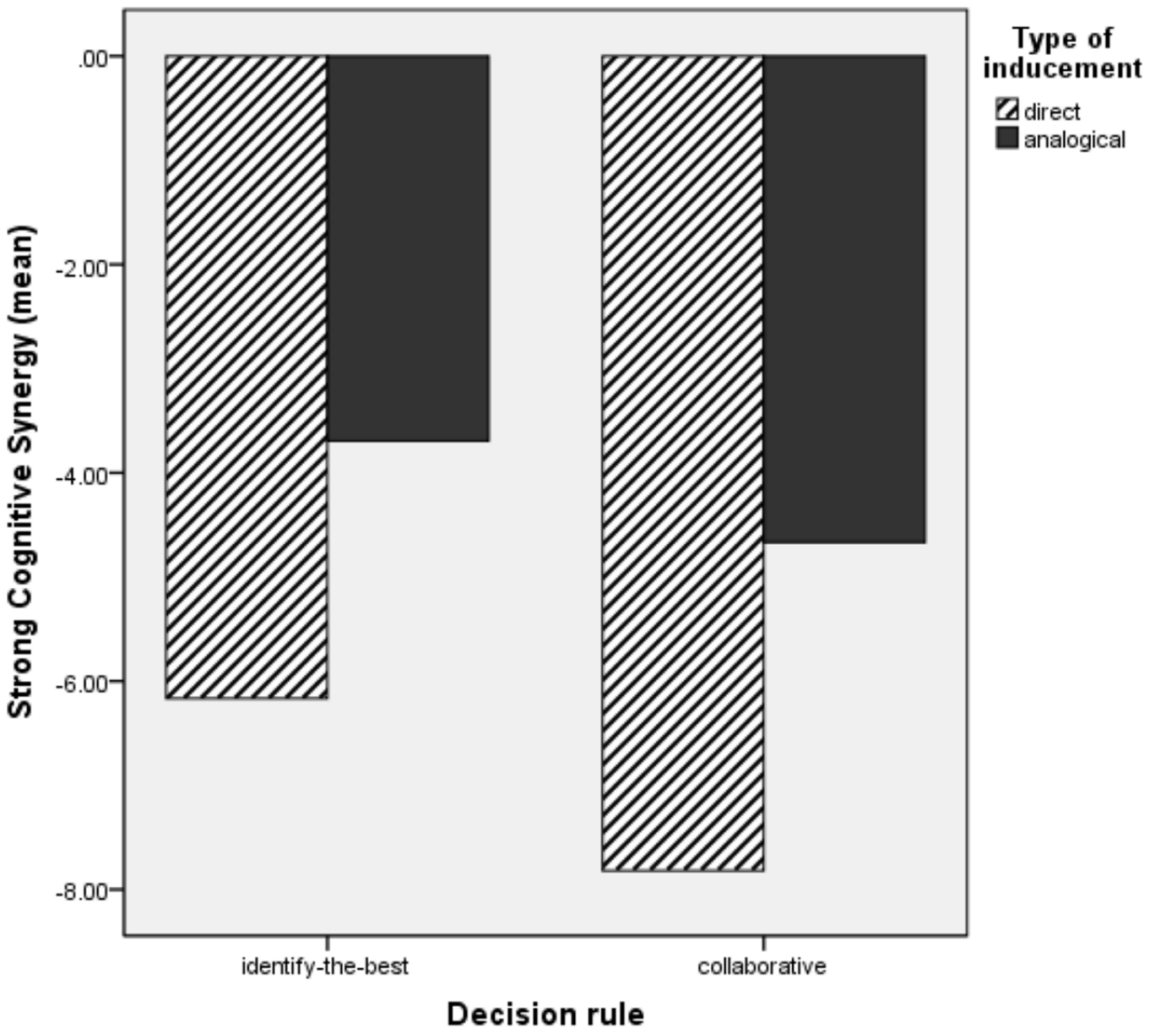
The interaction of decision rule and manipulation inducement on strong cognitive synergy Study 1.

The results of our first study do not provide empirical evidence for our hypotheses. No significant differences have been found between the identify-the-best decision rule and the collaborative rule or between the direct and the analogical type of inducement. When looking at descriptive statistics as well as Figure 1 and 2 we further identify that contrary to our expectations, groups perform better in the analogical manipulation than in the direct manipulation, irrespective of the type of rule followed, for both weak and strong cognitive synergy. Interestingly, for weak cognitive synergy groups manage to reach absolute levels of synergy (scores are positive) only in the analogical manipulation, again irrespective of the type of rule followed. This is not the case however for strong cognitive synergy, where synergy in absolute terms is not being reached in any of the four conditions. Our initial prediction was that groups following directly induced rules will outperform groups following analogical induced rules which involves an extra step in the process of establishing the group decision rules. One alternative explanation for this counterintuitive observation is that participants in the analogical conditions have more autonomy in defining their own decision rule, while groups with the direct rule manipulation have to follow an imposed decision rule. Groups that have a choice (high degree of autonomy) in defining their own working strategy are more committed to it and less prone to change it in a subsequent task [42]. Thus, the superiority of the analogical condition observed could be due to the fact that group members have a perception of responsibility for finding a successful decision rule and ultimately are more committed and involved in solving the decision task [42], [43]. In order to clarify whether this alternative explanation is supported by our unexpected observations in Study 1, we have designed a second study in which we contrast four conditions. The first two conditions (self-selection) are the baseline conditions in which groups are allowed to decide their own rule: (1) uninformed self – selection: no decision rule, groups are free to select any decision rule and no further influence is being exerted on the groups (USS) and (2) informed self-selection: groups are free to develop their own decision rule with the ultimate goal of becoming better than their best performing group member (ISS). These first two conditions are considered as baseline for refuting the self-selection explanation because in these conditions groups can decide what strategy to use thus should be more committed to it and more involved in solving their task. The last two conditions are induced decision rules selected from Study 1: CD and IBA. The goal of the second study is therefore to compare the two induced decision rule situations (CD and IBA) with the two self-selected conditions (ISS and USS). If the group's ability to reach cognitive synergy depends on the degree of autonomy in choosing a decision rule then the self-selection conditions should yield superior synergetic effects as compared to the induced decision rule.

## Study 2

### Methods

#### Participants and procedure

Three-hundred-thirty-three students enrolled in an introductory course at a Dutch University (Age_ mean_ = 19.09, 149 females) participated in the study. The students were informed that they will participate in a decision-making exercise as part of their collaborative learning experience and were debriefed at the end of the exercise. We have used a similar task as in Study 1, namely the NASA Moon Survival exercise [37]. The participants were asked to imagine that they are members of a space crew on a ship which has just crashed 200 miles from the meeting point with the mother-ship on the moon. Being left with only 15 intact items from their ship (e.g. matches, food) they had to decide which are the most important for their survival. Therefore, the task of the participants was to rank-order the 15 items from the most to the least important for their survival. In a first step, group members had to rate the objects individually. Next, the students have been assigned to 79 groups (average group size = 4.01) and were asked to perform the same task in groups. Similar to study 1, at the end of the task participants compared their individual performance scores with the group scores and reflected upon the impact of decision rules upon group dynamics.

#### Manipulations

In this second study, we compared two baseline conditions: uninformed self-selection (USS) and informed self-selection (ISS) with two conditions selected from the previous study: collaborative direct (CD) and identify-the-best analogical (IBA). We have used a between-group design. The 79 groups have been allocated to one of the four conditions: 21 groups to the USS condition, 21 groups to the ISS condition, 18 groups to the CD condition and 19 groups to the IBA condition. Condition CD and IBA have been induced similarly as in Study 1. In the USS condition, groups have been given no indication on how to decide as a group while in condition ISS groups have been instructed to design their own decision rule for 15 minutes while having in mind that the ultimate goal of the group is to become better than the best performing individual in the group. After designing the rule, groups have been asked to employ it as their strategy for the NASA group task. We have used the same measurement for the dependent variable including individual scores, group scores, weak and strong synergy as in Study 1.

### Results

In order to test our hypotheses we have ran a GLM analysis with strong and weak cognitive synergy as dependent variables. Similar to Study 1, group size, gender variety and the maximum score in the group have been used as control variables. Descriptive statistics and correlations between the variables included in the analysis are presented in Table 3. Descriptive statistics of the manipulations are presented in Table 4.

**Table 3:** Correlation table with descriptive statistics Study 2 (N = 79).

	Mean	*SD*	1	2	3	4
1.Weak cognitive synergy	9.80	8.76				
2.Strong cognitive synergy	0.01	9.63	0.84***			
3. Gender variety	0.36	0.31	−0.11	−0.08		
4. Individual maxim score	51.01	8.23	−0.04	−0.4***	0.07	
5. Group size	4.01	1.03	0.15	−0.02	−0.12	0.12

Note: ***<.01.

**Table 4:** Descriptive statistics manipulations Study 2.

	Mean		SD		N
	WS	SS	WS	SS	WS/SS
Uninformed self-selection	7.63	−2.47	8.41	9.33	21
Informed self-selection	9.99	−1.36	8.12	8.87	21
Identify-the-best analogical	11.14	3.05	7.97	9.80	19
Collaboration direct	10.71	1.38	10.79	10.34	18

Note: WS =  weak cognitive synergy; SS =  strong cognitive synergy; N =  number of groups.

**Figure 3:**
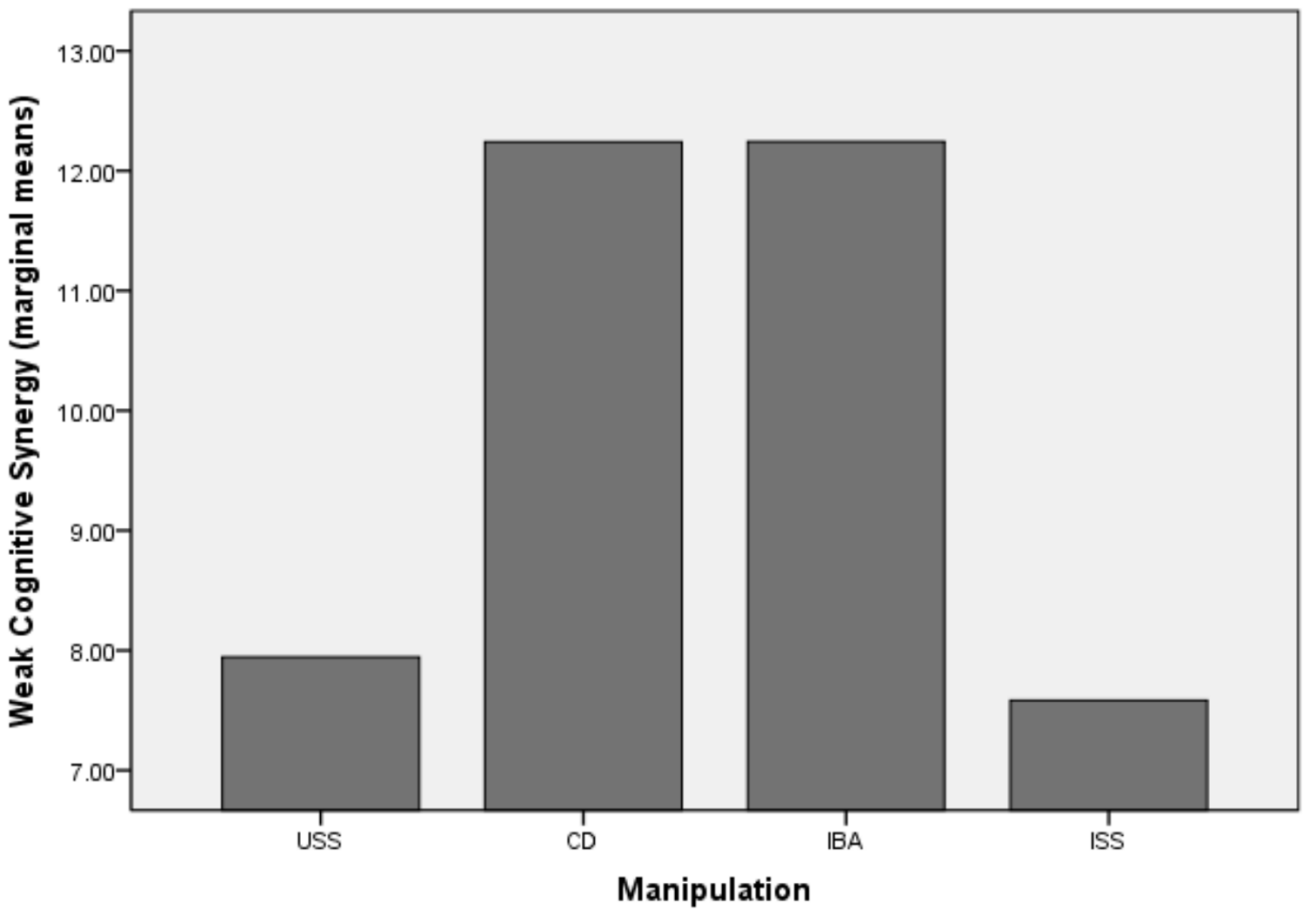
The impact of manipulations on weak cognitive synergy Study 2.

**Figure 4:**
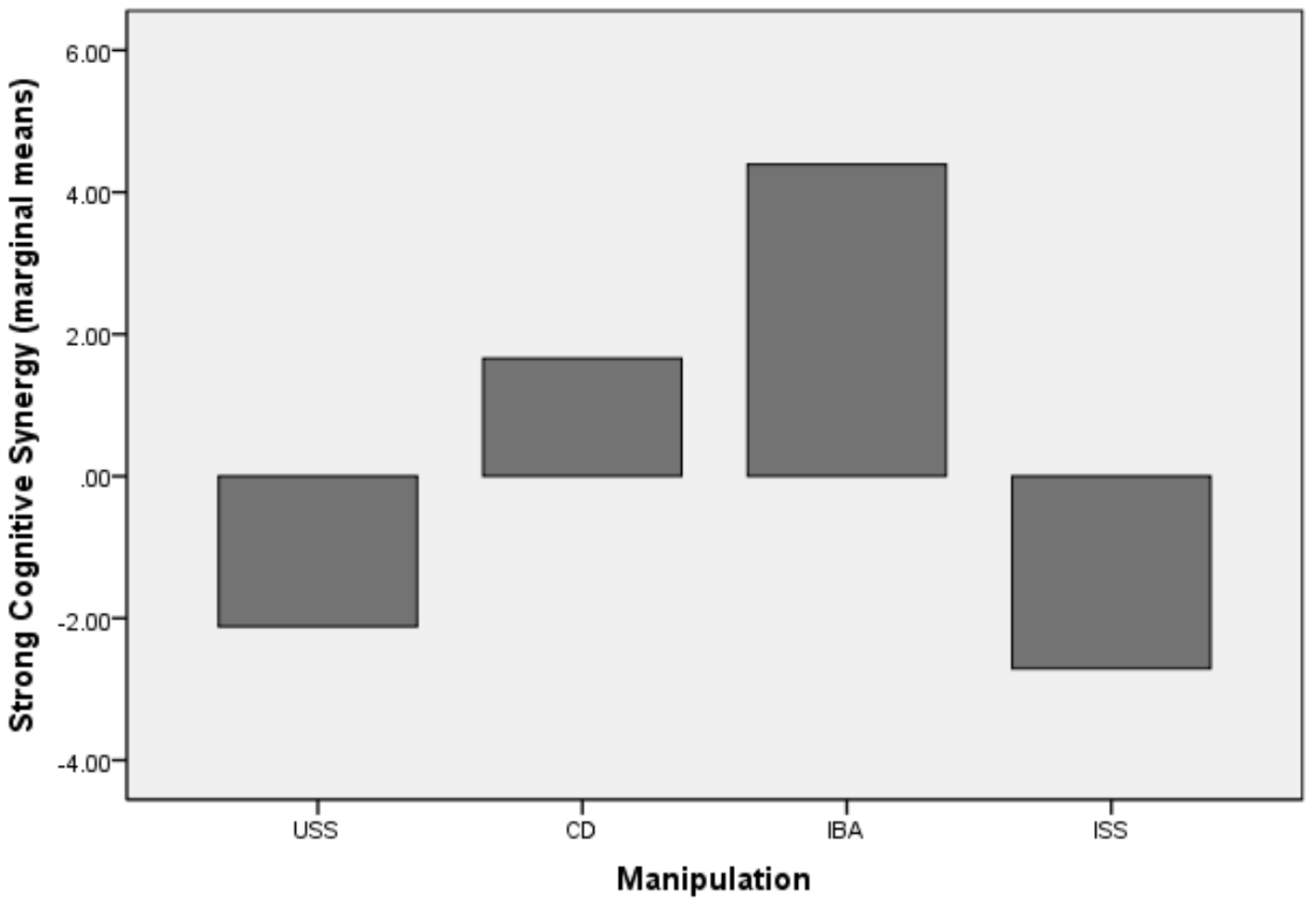
The impact of manipulations on strong cognitive synergy Study 2.

Our results indicate no overall effect of the manipulation upon strong cognitive synergy, F (1, 79) = 2.31, p = 0.08 or weak cognitive synergy with F (1, 79) = 1.33, p = 0.27. The maximum score in the group had a significant effect on strong cognitive synergy F (1, 79) = 14.57, p = 0.00, with a partial *η2* = .16 and observed power *π* = .96 and no effect on weak cognitive synergy, with F (1, 79) = 0.18, p = 0.66. For weak cognitive synergy, subsequent t-test contrasts indicate no significant mean difference between any of the four conditions. For strong cognitive synergy however, a significant mean difference has been identified between the USS (M = −2.47, SD = 9.33) and IBA (M = 3.05, SD = 9.80), t = 6.51, p = 0.02, CI [0.88; 12.13] as well as a significant difference between the ISS (M = −1.36, SD = 8.87) and IBA, t = 7.10, p = 0.03, CI [0.63; 13.56]. The comparison of conditions is also displayed in Figure 3 and 4.

## General Discussion

The results of our first study were not conclusive with respect to the influence of decision rules on group cognitive synergy. One plausible explanation is the small sample size. However, descriptive statistics indicate, contrary to our expectations that the analogical rule appears to be more efficient than the direct one. In order to check what explains the superiority of the analogical inducement we have designed a second study in which we ruled out the degree of group autonomy and involvement in choosing a decision rule as an alternative explanation for our observations. In the analogical manipulation, groups have a large degree of autonomy with respect to the decision rule they have to define and follow when making a decision. Groups are instructed to define their own decision rule by making analogies with successful rules inferred from the scenarios that point towards collaboration or identify-the-best decision rules. In the directly induced decision rules, the degree of autonomy in using a particular decision rule is restricted as groups are being instructed to follow either a collaborative rule or an identify-the-best rule. In previous research the degree of autonomy has been linked with the perception of responsibility for one's decision and commitment to the task, in such a way that higher degree of autonomy leads to higher commitment and responsibility [42], [43]. If the higher synergy achieved in the analogically induced conditions were to be explained by the larger degree of autonomy, then in the second study, the two free decision rule conditions (ISS and USS) should have outperformed the CD and IBA. However, the results of the second study rule out this alternative explanation. Groups in the IBA condition (with an analogical decision rule induction) outperformed groups that are given the freedom to choose their own decision rule, with or without the explicit goal of becoming better than their best performing group member (ISS and USS).

The current paper has several contributions, both theoretical and practical. First, we contribute to the decision-making stream of research by indicating the beneficial effects of a heuristic decision rule (imitate-the-successful/analogical inducement) on decision quality. This type of inducement proves to be a stronger manipulation than the content of the rule in itself and thus practitioners should further consider not only the decision rule used to stimulate groups to perform better than their best performing individual member, but also the way in which this decision rule is being communicated and induced.

Next to the overall beneficial effect of the analogical inducement, the combination of two heuristics (imitate-the-successful and identify-the-best) proved to be the most beneficial for groups in their attempt to achieve strong cognitive synergy. Groups following simple heuristics that are adapted to the contexts in which they operate, arrive at decisions that outperform the ones reached by groups relying on rather unclear strategies. Identifying the best performing group member and improving his/her performance is a decision rule particularly adapted for strong cognitive synergy, given that the core of strong synergy lies in groups outperforming its best individual member. Our results are also indicative of the less-is more effect [29] in group settings, given that groups following an identify-the-best decision rule perform slightly better than groups following a collaborative rule. This is consistent with simulation studies indicating the superiority of the expert rule (where the expert person in the group is identified and followed) relative to the aggregate rule (information pool from multiple individuals) [19].

Second, we contribute to the cognitive synergy literature. While groups are widely employed in organizations with the assumption that their performance should exceed the performance of their individual members, empirical evidence shows that this is rarely the case [11], [44]. Our findings indicate that strong group synergy is more likely to be achieved when groups (1) follow analogically induced decision rules rather than directly induced rules (2) follow the identify-the-best decision rule (induced analogically) rather than self-selected rules. This finding has practical implications for group interventions. Groups that were instructed to self-select their own rule displayed the weakest performance, while the highest synergy was obtained when groups used the CD and the IBA decision rule. Groups in the IBA condition in the second study were the only ones that managed to reach real levels of strong cognitive synergy. Therefore, our study comes with suggestions on what types of strategies are useful for decision-making groups that struggle to increase their performance and perform better than their best individual member.

### Limitations and directions for further research

Next to its contributions, our study has also certain limitations. First, the sample size used (especially in the first study) is rather small, a limitation inherent to experimental studies with group level manipulations. Our non-significant results between the analogical and the direct condition could be explained by the small sample size. Further studies should try to replicate these results and check the generalizability of our results on different other (larger) samples. Second, the task type used in our experimental studies is a boundary condition for the superiority of the analogical decision rule. We have tested the efficiency of such a rule in a decision-making task where the decision quality reflects how much the decision is aligned with an expert's decision. Drawing from previous experiences of successful groups fits well with the type of task groups have to accomplish. It could be the case that in other types of tasks (e.g. creativity or judgmental tasks) different decision strategies are also effective in achieving cognitive synergy. Therefore, further studies should explore the fit between the type of task and the type of decision rules as an important antecedent of group cognitive synergy. Finally, in the first study we did not control for the effect of time spent on task on strong and weak cognitive synergy. Nevertheless, based on the effects reported in Study 2 we can disentangle the effect of extra time as both IBA and ISS conditions had extra time allocated to prepare the task, yet the difference between the two is significant. This pattern of results is in line with previous studies [8] showing that the effect of the normative framework used by groups qualified the effect of time spent on task on the quality of group decision.

While following Kurt Lewin's logic [18], the way we attempted to change the groups as systems by inducing several decision rules generated interesting insights into the impact of decision rules on strong group synergy. The analogical induction seems to yield the potential for generating strong synergy in decision-making groups. Although analogy-making proves to be a useful tool in a large array of social contexts, the number of studies investigating how analogies work in groups and their functions are rather scant [45]. Further studies should explore the role of analogies and analogical thinking in groups together with the mechanisms that explain the superiority of the analogical decision rules. One interesting avenue of research here could be to connect this type of rule inducement with heuristic decision-making, such as the imitate-the-successful one and shed some light on why this particular type of heuristic proves to be the most adaptive for groups.
